# Validation of microRNA-199b as A Promising Predictor of Outcome and Response to Neoadjuvant Treatment in Locally Advanced Rectal Cancer Patients

**DOI:** 10.3390/cancers13195003

**Published:** 2021-10-05

**Authors:** Ion Cristóbal, Andrea Santos, Jaime Rubio, Cristina Caramés, Sandra Zazo, Marta Sanz-Álvarez, Melani Luque, Juan Madoz-Gúrpide, Federico Rojo, Jesús García-Foncillas

**Affiliations:** 1 Cancer Unit for Research on Novel Therapeutic Targets, Oncohealth Institute, IIS-Fundación Jiménez Díaz-UAM, E-28040 Madrid, Spain; andrea.santos@quironsalud.es (A.S.); jaime.rubiop@quironsalud.es (J.R.); ccarames@fjd.es (C.C.); 2 Translational Oncology Division, Oncohealth Institute, IIS-Fundación Jiménez Díaz-UAM, E-28040 Madrid, Spain; 3 Medical Oncology Department, University Hospital “Fundación Jiménez Díaz”, UAM, E-28040 Madrid, Spain; 4 Pathology Department, IIS-Fundación Jiménez Díaz-UAM, E-28040 Madrid, Spain; szazo@fjd.es (S.Z.); marta.sanza@quironsalud.es (M.S.-Á.); melani.luque@quironsalud.es (M.L.); jmadoz@fjd.es (J.M.-G.); frojo@fjd.es (F.R.)

**Keywords:** MiR-199b, locally advanced rectal cancer, prognosis, pathological response

## Abstract

**Simple Summary:**

The clinical management of locally advanced rectal cancer (LARC) patients would benefit for the establishment of molecular markers that could anticipate the response to neoadjuvant chemoradiotherapy (CRT). We aimed here to validate in an independent cohort our previous findings about the prognostic value showed by miR-199b in LARC as well as to explore its status in the disease progression. Notably, we confirmed in this work that miR-199b has a predictive value of both outcome and response to preoperative CRT in LARC patients further strengthen its potential usefulness in this disease.

**Abstract:**

The absence of established predictive markers with value to anticipate response to neoadjuvant 5-fluorouracil (5-FU)-based chemoradiotherapy (CRT) represents a current major challenge in locally advanced rectal cancer (LARC). The tumor suppressor microRNA (miR)-199b has been reported to play a key role determining 5-FU sensitivity of colorectal cancer cells through the regulation of several signaling pathways, and has emerged as a novel molecular target to overcome the 5-FU resistant phenotype. Moreover, miR-199b downregulation was described as a common alteration that predicts lack of response to preoperative CRT in LARC but this issue needs to be confirmed in independent larger cohorts. Here, we evaluate the clinical impact of miR-199b in LARC and perform additional analyses to further clarify its potential relevance as novel marker in this disease. Thus, miR-199b expression was quantified by real-time-PCR in a cohort of 185 LARC patients, observing this miR downregulated in 22.2% of cases and significantly associated with higher tumor size (*p* = 0.026) and positive lymph node after CRT (*p* = 0.005), and higher pathological stage (*p* = 0.004). Notably, this alteration showed a strong independent predictive value of poor pathological response to neoadjuvant CRT (*p* = 0.004). Moreover, the subgroup of cases with low miR-199b levels had a markedly shorter overall (*p* < 001) and event-free survival (*p* < 0.001), and multivariate analyses showed that miR-199b deregulation represents an independent prognosticator for patient outcome in LARC. Interestingly, the prognostic impact of this miR was strongly significant in both younger and elderly patients, and was very effective determining patient recurrence (*p* = 0.004). Finally, we compared miR-199b expression profiles in a set of cases with pre and post-treatment samples available, observing that only a minimal response leads to miR-199b increase levels, further suggesting its potential clinical and therapeutic relevance as a promising marker and novel molecular target for the management of LARC.

## 1. Introduction

Colorectal cancer (CRC) is the fourth most commonly diagnosed cancer worldwide (10%), and the second leading cause of cancer death (9.4%). In Spain, the total number of new cases is estimated to be more than 44,000 each year, and reaches around 14.6% of total deaths from cancer annually [1,2]. Approximately, almost one-third of all newly diagnosed CRCpatients have rectal tumors [3]. Of note, locally advanced rectal cancer (LARC) has been progressively considered and treated as an independent disease due to several factors such as its primarily extra peritoneal location, high local recurrence risk and differences in metastatic behavior [3,4,5]. Over the last few decades, several studies have explored different treatment strategies to improve the outcome of LARC patients, leading to significant changes in the clinical management of this disease [6]. The current standard treatment recommended for LARC patients is referred to a multimodal neoadjuvant treatment consisting of a long-course preoperative 5-fluorouracil (5-FU)-based chemoradiotherapy (CRT) and short-course preoperative radiotherapy (SCPRT) followed by total mesorectal excision (TME) [7,8]. This therapeutic strategy has improved the management of LARC patients and was established after numerous studies demonstrating that in patients with resectable rectal cancer, SCPRT or CRT before TME significantly leads to a lower local relapse rate and better response than TME followed by adjuvant CRT or TME alone [9,10,11]. With the implementation of total mesorectal excision surgery and neoadjuvant chemoradiotherapy (nCRT), local recurrence rates have markedly declined and complete pathological response (pCR) is observed in more than 20% of cases [11]. However, due to the high morbidity risk of the surgery and the severe side effects derived by the treatment [7,12], it is necessary to identify patients who will benefit from this treatment. One of the most debated treatment approaches on LARC management in the recent years is the non-operative “watch and wait” strategy that aims for organ preservation, which will highly improve the quality of life in patients with complete clinical response to neoadjuvant therapy [7,13]. Currently, there is a lack of well-established biomarkers that can reliably predict pathological response to preoperative CRT, which will be of crucial importance since it would improve patient outcomes as well as reduce morbidities and allow designing more effective therapeutic strategies for the subgroup of predicted non-responder LARC patients. In this context, microRNAs (miRs) have progressively emerged in the last decade as promising candidates for predicting therapy response in LARC, due to their stability, facile detection and disease-specific expression in human tissue and blood [14,15]. 

MiRs are single-stranded and non-coding RNAs with small length (around 18–25 nucleotides), that play their main function at the post-transcriptional level through the induction of the degradation and repression of specific target genes. These molecules are involved in the regulation of many biological processes, such as cell proliferation, cell differentiation, apoptosis and embryonic development. Additionally, growing evidence has demonstrated that deregulation of miRs contributes to tumorigenesis and tumor progression in a wide variety of tumor types, including rectal cancer, acting as oncogenic or tumor suppressor miRs, depending on the targeted mRNAs. Deregulation of miRs could be caused by multiple mechanisms, such as epigenetic alterations, chromosomal abnormalities, as well as changes in their transcriptional regulation [14,15,16,17,18]. Lately, increasing functional data of specific miRs, such as miR-199b, that play important roles in rectal cancer have been analyzed [19]. MiR-199b is a tumor suppressor largely involved in human cancer. Its downregulation has been associated with cancer progression and metastasis through the modulation of different signaling pathways in various tumor subtypes, such as breast cancer [20,21], medulloblastoma [22], prostate cancer [23], hepatocellular carcinoma [24,25] and acute myeloid leukemia [26]. In CRC, several studies have explored the significance of miR-199b. Thus, this miR has been reported to control CRC distant metastasis progression through the CREB/KISS1 [27] and DDR1/JAG1 [28] signaling pathways. In addition, our group described that miR-199b directly targets SET, a potent endogenous inhibitor of the tumor suppressor PP2A [29], thereby controlling the activation status of the SET/PP2A axis in CRC, and that its downregulation determines poor prognosis in both localized and metastatic CRC patients [30,31]. It has also been reported that the transcription factor E2F7 reduced the expression of miR-199b by binding to the miR-199b promoter, upregulating USP47 and MAPK, leading to an increase on the proliferation, invasion and migration abilities of colon cancer cells [32]. In rectal cancer, a recent study by Baek and colleagues showed that high expression of both miR-199a and miR-199b were associated with superior clinical outcomes in a cohort of 65 tissue and 89 serum samples [33], and our group has recently observed that miR-199b downregulation determines poor outcome and lack of response to preoperative CRT in a cohort of 82 LARC patients [34]. These observations are probably due to the role of the miR-199b as a key regulator of 5-FU response, which has been experimentally demonstrated in previous works by our group and others [27,32,34]. Thus, the alteration in miR-199b expression has emerged as a promising marker in LARC, but the short number of patients included in the studies reported to date makes necessary the validation of its clinical impact in independent larger LARC patient cohorts.

We aim here to validate the potential clinical relevance of miR-199b as a novel marker with prognostic value in a cohort of 185 LARCpatients. We observed that low miR-199b expression levels independently determined both poor outcome and lack of pathological response to preoperative CRT. Moreover, we observed that this alteration is able to predict patient recurrence and that its clinical impact is valid in both subgroups of younger and elderly cases. Finally, we will further explore the usefulness of miR-199b analyzing a set of LARC patients with paired pre and post-treatment material available, observing a clear correlation between miR-199b expression and disease progression in LARC patients.

## 2. Experimental Section

### 2.1. Patient Samples

Consecutive specimens from 185 LARC cases with enough material available could be selectedfor our validation study.Clinical information from patients histologically diagnosed of LARC and treated with preoperative CRT between 2006 and 2017 in the University Hospital Fundacion Jimenez Diaz (Madrid, Spain) was obtained by two oncologists from medical records. However, only 163 cases had enough clinical follow-up to be included for survival analyses. Magnetic resonance image of the pelvis and/or transrectal ultrasound was used to establish an accurate preoperative locoregional tumor staging. All patients included in this work were excluded for metastatic disease by a full body computed tomography. Preoperative CRT regimens were based on 5-FU and surgery was programmed 6 to 8 weeks after completion of neoadjuvant treatment. All participants gave written informed consent for tissue storage and analysis at Fundación Jiménez Díaz biobank, Madrid (Spain). This study was approved by the ethical committee and institutional review board of Fundacion Jimenez Diaz (ref. PIC202-20).

### 2.2. Determination of Pathological Response

Tumor samples from initial biopsies obtained by colonoscopy were evaluated following the criteria established by the College of American Pathologist guidelines for invasive carcinomas (TNM, 7th ed.). Two pathologists who were blinded to patient clinical dataused the modified Ryan classification to evaluateindependently tumor regression grade in each case, which was compared with the primary tumor [26]. Four levels of response are considered by this the modified Ryan classification: complete response that indicates no viable cancer cells (score 0), moderate response with single cells or little groups of cancer cells (score 1), minimal response that indicates residual cancer outgrown by fibrosis (score 2), and poor response in which the patient shows minimal or no tumor kill with extensive residual cancer (score 3).

### 2.3. Total RNA Isolation

The RecoverAll Total Nucleic Acid Isolation kit (Thermo Fisher Scientific, Waltham, MA, USA) we used to perform total RNA extraction from formalin-fixed paraffin-embedded (FFPE) tumor specimens, according to manufacturer’s protocol. Quantification and quality evaluation of the RNA obtained by the extractions wascarried out using a NanoDrop Spectrophotometer (Thermo Fisher Scientific, Waltham, MA, USA).

### 2.4. Quantification of miRNA Expression Levels

Reverse transcription of the tumor samples was performed using the TaqManH MicroRNA Reverse Transcription Kit (Applied Biosystems Waltham, MA, USA). Specific TaqMan MicroRNA Assays(AppliedBiosystems) for miR-199b (reference number: 000500) and U6B (reference number:_001093) as internal control were used to quantify mature miRs by quantitative real-time reverse transcription polymerase chain reaction (QRT-PCR) in anApplied Biosystems 7500 Sequence Detection System. Experimental conditions: 95 °C for 10 min, followed by 45 cycles of 95 °C for 15 s, and 60 °C for 1 min. Analysis of relative gene expression data was carried out using the ∆*C*T method [27]. MiR-199b downregulation was considered when the expression in a sample was lower than the mean minus standard deviation (SD) of the patient cohort, as previously described [24].

### 2.5. Statistical Analysis

All statistical analyses were carried out using the software tool SPSS v20 for windows (SPSS Inc, Chicago, IL, USA) and GraphPad Prism v7.0 (GraphPad Software Inc, San Diego, CA, USA). Associations between miR-199b and clinical and molecular parameters were studied applying the Chi-Square test based on bimodal distribution of data. Kaplan–Meier survival analyses were performed using the log-rank test for the evaluation of both overall survival (OS) and event-free survival (EFS). OS was calculated as the time length from patient diagnosis to death or last follow-up, and EFS as the time length from diagnosis to local or distant recurrence, death or last follow-up.For multivariate analyses we used the Cox proportional hazards model including all significant parameters in the univariate analyses. Comparisons between tumor samples from each patient before and after preoperative CRT were performed using Mann–Whitney and paired t tests. This study has been performed following the Reporting Recommendations for Tumor Marker Prognostic Studies (REMARK) guidelines [28]. Statistical significance was considered when *p*-value was lower than 0.05.

## 3. Results

### 3.1. MiR-199b Is Downregulated in a Subgroup of Patients and Associates with Molecular and Clinical Parameters

We quantified the expression levels of miR-199b in a cohort of 185 LARC patients in order to analyze the prevalence of miR-199b downregulation, observing this alteration in 22.2% of cases (41 out of 185). We found no association of miR-199b downregulation with patient age, ECOG status or stage in this patient cohort (Table S1). After excluding 22 patients from the initial cohort due to a lack of enough clinical follow-up data available in the medical records for those cases, we evaluated the potential association of miR-199b expression with clinical and molecular parameters in a series of 163 LARC patients from our cohort with clinical follow-up data available. 

Notably, miR-199b downregulation significantly correlated with higher tumor size after CRT (*p* = 0.026), positive lymph node after CRT (*p* = 0.005) and advanced pathological stage (*p* = 0.004). The analysis of the association between miR-199b expression and molecular and clinical parameters of the patient cohort studied are shown in Table 1.

In order to establish the patient subgroups of both tumor size and lymph nodes associated with miR-199b expression, we stratified our cohort considering these parameters. Of interest, we found that miR-199b downregulation was strongly associated with those cases with tumor size 2 or higher (*p* = 0.007) and those with presence of lymph node metastases (*p* = 0.002) (Table S2).

### 3.2. MiR-199b Downregulation Determines Poor Pathological Response to Neoadjuvant CRT in Locally Advanced Rectal Cancer Patients

We next evaluate the usefulness of miR-199b expression levels to predict pathological response to preoperative CRT in our LARC patient cohort. Firstly, we stratified our cohort of 163 cases in responders (*n* = 79) and non-responders (*n* = 84), and observed that the subgroup of cases with miR-199b downregulation was strongly associated with lack of response (71.8% vs. 28.2%, *p* = 0.004) (Table 2).

To further confirm the predictive value of response to neoadjuvant CRT of miR-199b expression levels, we carried out a multivariate analysis, observing that preoperative low miR-199b expression is an independent predictor of response to neoadjuvant CRT in our cohort of 163 LARC patients (*p* = 0.007) (Table 3).

### 3.3. MiR-199b Downregulation Is an Alteration That Predicts Poor Outcome and Recurrence in Locally Advanced Rectal Cancer Patients

**Figure 1:**
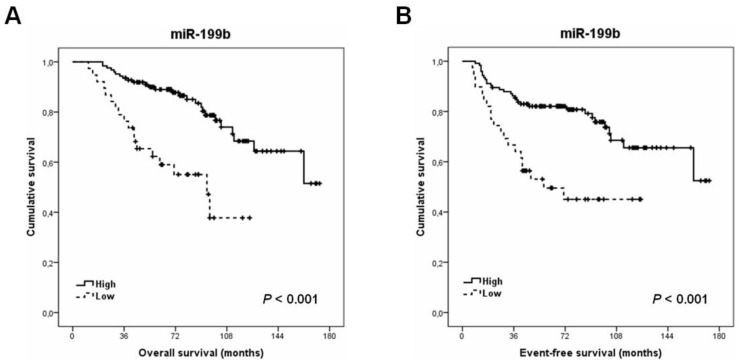
Clinical impact of miR-199b expression in LARC patients. Kaplan–Meier analyses for: (**A**) overall and (**B**) event-free survival in a cohort of 163 LARC cases.

To evaluate the clinical significance of miR-199b downregulation in LARC, we next studied the potential impact of this alteration determining patient outcome in this disease. We analyzed all the 163 cases with clinical follow-up data available to perform survival analyses. The patient cohort included 95 male and 68 female, with a median of age of 69 years and an age range from 36 to 86 years. Interestingly, the results obtained in this analysis showed that the subgroup of patients with low miR-199b expression had a markedly shorter OS compared to those cases with high miR-199b levels (139 versus 82 months, *p* =< 0.001) (Figure 1A). Moreover, we also found that miR-199b downregulation determined poorer EFS in our patient cohort (132 versus 73 months, *p* =< 0.001) (Figure 1B).

Notably, we performed a multivariate analysis showing that low miR-199b expression represents an unfavorable independent predictor of both OS and EFS in our cohort (Table 4 and Table S3).

**Figure 2:**
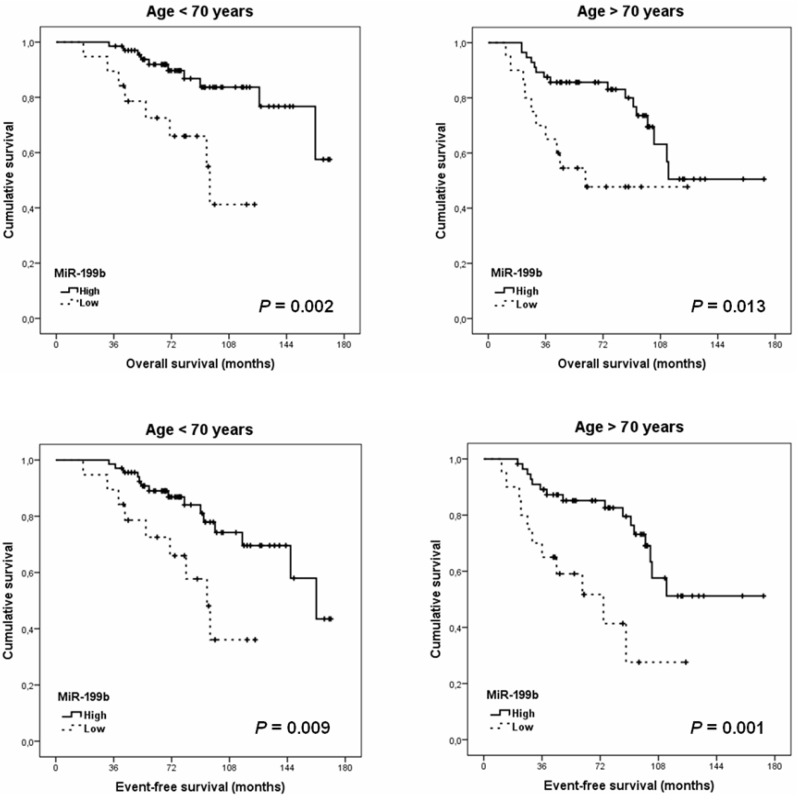
Kaplan–Meier analyses showing the prognostic impact of miR-199b expression predicting OS and EFS in LARC patients stratified by age.

We alsoinvestigated if the prognostic impact of miR-199b could be affected by patient age. This analysis showed that the prognostic value of this miR remained significant in both subgroups of patients younger (*p* = 0.002 for OS; *p* = 0.009 for EFS) and older than 70 years (*p* = 0.013 for OS; *p* = 0.001 for EFS), which indicates that miR-199b downregulation determines poor outcome in LARC patients with independence of their age (Figure 2).

In order to assess the potential involvement of miR-199b deregulation in the disease progression, we next investigated its association with patient recurrence. Interestingly, we found that the subgroup of patients with low miR-199b expression showed markedly higher recurrence rates compared with those with high miR-199b levels (*p* = 0.004) (Table 5). Moreover, we analyzed if miR-199b downregulation was associated with the metastatic niche in 31 cases that progressed to metastatic disease with clinical data available, butmiR-199b did not show any association with the place of relapse (*p* = 0.543) (Table 5).

To further clarify the clinical relevance of miR-199b in the disease progression and due to the deregulation of this miR was strongly associated with recurrence, we evaluated its potential value as a predictor of time to metastatic progression. However, the presence of this alteration did not determine a shorter time to progression (*p* = 0.599) (Figure S1).

### 3.4. MiR-199b Retains Its Downregulated Levels in Post-Treatment Samples from Those Cases with Lack of Response to Neoadjuvant CRT

To further evaluate the status of miR-199b in the disease progression, we analyzed the expression profile of this miR in a set of paired pre- and post-treatment patient samples from our cohort. Of note, we considered the term “treatment” as the neoadjuvant CRT received by the LARC patients included in this study. Obviously, we could quantify the miR-199b expression only in cases that did not respond (RYAN 2 and 3), since those who showed pathological response had a lack or not enough tumor material in the post-treatment specimens to perform this analysis.

Thus, we compared miR-199b expression of in a set of 24 LARC patients with paired pre- and post-treatment tumor samples available. We found similar miR-199b levels between the subgroups of pre and post preoperative CRT (*p* = 0.690) (Figure S2A). MiR-199b expression was found slightly increased with a fold change of 1.22 in post vs. pre-treatment specimens. The scatter plot analyzing the changes in the expression levels of this miR in each case confirmed the lack of differences (*p* = 0.666) (Figure S2B).

**Figure 3:**
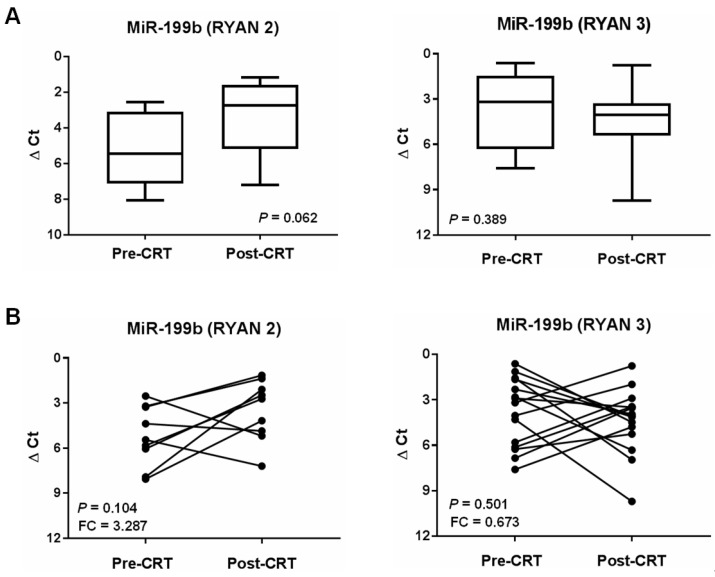
MiR-199b expression profile during LARC progression: (**A**) box plots and (**B**) scatter plots comparing miR-199b expression in pre-treatment and post-treatment samples from a series of 24 LARC patients.

As we could not evaluate the group of responder cases by the lack of tumor material to quantify the levels of miR-199b, we next hypothesized that we could observe differences in the expression of this miR if we stratified our set of non-responders by their RYAN score since we evaluated RYAN 2 (*n* = 9) together with RYAN 3 patients (*n* = 15) in our first analysis. Notably, we found a 3.29-fold increase in miR-199b expression in the post-treatment samples from RYAN 2 patients, but significance was not achieved by the low number of cases analyzed (*p* = 0.062) (Figure 3A). However, we observed that miR-199b levels decreased in post-treatment samples from those cases with RYAN 3 with a fold change of 0.67. Similar results were observed in the analysis of changes in each patient using scatter plots (Figure 3B).

## 4. Discussion

The urgent need for robust and validated markers to anticipate response to preoperative CRT in LARC patients currently remains to be addressed. Considering this issue and the promising results obtainedby our group with the miR-199b in this disease, we prompted to validate our previous observations in an independent larger LARC cohort as well as to perform additional analyses to fully clarify the clinical impact and potential usefulness of this miR. 

Here, we have observed miR-199b downregulated in 22.2% of cases, which is concordant with the prevalence described in our previous work with LARC patients (26.4%) [34], and differences could be probably due to the low number of LARC cases analyzed in that work (*n* = 110) compared to the current study (*n* = 185). Moreover, we had reported a prevalence of 21.6% and 24.7% in early stage and metastatic CRC patients [30,31], respectively. In both studies, patients were stratified by the site of primary tumor and miR-199b downregulation was found in 20% of rectum tumors vs. 26.4% of colon tumors in patients with metastatic disease, and in 18.6% vs. 22% in patients with localized disease at diagnosis. These observations would suggest that miR-199b downregulation is an alteration that could have a slight higher prevalence in colon than in rectal cancer patients, with independence of the progression of the disease. However, these results have to be confirmed in forthcoming studies. Of note, we have described in the present work the association of low miR-199b expression with lymph node positivity as well as high pathological stage, observations that are similar to previous studies of miR-199b in LARC patients [33,34]. However, the association with higher tumor size observed here (Table 1) did not achieve significance in the previous work and the explanation could be the higher number of cases evaluated in the current study (163 vs. 82). In fact, that significant association between miR-199b and tumor size was observed when patients were grouped in low (pyT = 0–1) and high tumor size (pyT > 1), which was also confirmed here (Table S2).

The confirmation of the predictive value of response to neoadjuvant CRT of miR-199b (Table 2) represents a very relevant result of this study due to the urgent need for robust markers for this fact in the LARC clinical management. Notably, the results showed here are in total concordance with the published in the last work of our group about LARC and miR-199b, and the multivariate analyses highlighted its independent clinical impact (Table 3). We have previously reported the role of miR-199b promoting 5-FU response in a SET-dependent manner leading to the reactivation of the tumor suppressor PP2A [31,34]. However, other studies in the literature have demonstrated that miR-199b increases 5-FU sensitivity of CRC cells through the regulation of the SIRT1/CREB/KISS1 as well as DDR1/JAG 1 signaling pathways [27,28]. Therefore, these observations could explain that this alteration serves as a marker of poor outcome in CRC independently of SET [30], and are in concordance with our results showed here about the clinical impact of miR-199b as a predictor of pathological response to neoadjuvant CRT in LARC patients. 

Furthermore, we also analyzed the prognostic value of miR-199b in our cohort of 163 LARC patients and observed that its downregulation confers a very poor outcome (Figure 1), which validates the previous findings indicating that low miR-199b expression represents a marker of shorter overall and event-free survival in LARC patients [34]. As an important consideration, the potential relevance of the patient age as a parameter that could affect the prognostic impact of miR-199b had not been previously evaluated in LARC. However, several works in the literature with localized and metastatic CRC patients have shownthat miR-199b downregulation only achieve statistical significance as predictor of shorter progression-free survival in the subgroup of patients younger than 70 years [30,31]. Of note, we analyzed here the prognostic impact of miR-199b expression after stratifying our cohort by age, and observed that both subgroups of younger and elderly patients had similar statistical significance in the survival analyses performed (Figure 2). These results would suggest that there are no differences in the clinical impact of LARC patients by age. Therefore, the differences previously described in early stage CRC [30] could be higher in the subgroup of colon cancer compared to rectal cancer patients. This issue needs to be clarified in forthcoming studies with series including colon and rectal cancer patients and analyses of them separately.

The involvement of miR-199b deregulation in disease progression has been previously reported in CRC. Shen and colleagues observed miR-199b downregulated in hepatic metastasis tissues from CRC patients [27], which was also described by our group [31] in metastatic CRC. These observations prompted us to analyze its potential involvement in disease progression also in LARC, an issue that remained unexplored. Notably, our results here show a strong association between miR-199b downregulation and recurrence (Table 5), which is in concordance with the results described by Shen and colleagues in their work and highlight the relevance of this alteration also in LARC. Finally, the results obtained with the comparative analysis between samples pre and post-CRT (Figure 3) further strengthen the role of miR-199b in the progression of LARC and its potential usefulness as marker for the clinical management of this disease. However, several limitations of our study have to be taken into consideration. Thus, the validation of these findings in a larger and multi-centric cohort of LARC patients would strengthen the clinical relevance of miR-199b in this disease. Moreover, the potential usefulness of this miR as a circulating marker needs to be confirmed in forthcoming studies. Finally, the potential dependence between miR-199b expression and ethnic characteristics has not been evaluated and remains to be clarified.

## 5. Conclusions

Altogether, we show here that miR-199b downregulation is a common event in LARC that determines poor pathological response to neoadjuvant CRT and defines a subgroup of patients with markedly shorter overall and event-free survival in both subgroups of younger and elderly patients. Moreover, our study provides novel relevant findings about the clinical status of this miR in the disease progression, showing a strong association with patient recurrence. Notably, we also found that the changes in miR-199b expression during the disease progression correlates with the grade of response showed by the patients, which further suggest the relevance of miR-199b determining response to 5-FU-based therapies.

## Figures and Tables

**Table 1:** Association between miR-199b expression with clinical and molecular characteristics in a cohort of 163 LARC patients.

Parameter	No. Cases	No. miR-199b High (%)		No. miR-199 Low (%)		*p*
MiR-199b	163	124	(76.1)	39	(23.9)	
Gender	163	124		39		0.112
Male	95	68	(71.6)	27	(28.4)	
Female	68	56	(82.4)	12	(17.6)	
Age	163	124		39		0.504
<70	87	68	(78.2)	19	(21.8)	
>70	76	56	(73.7)	20	(26.3)	
ECOG ^1^	163	124		39		0.634
0	112	84	(75)	28	(25)	
1–2	51	40	(78.4)	11	(21.6)	
Clinical stage pre-CRT ^2^	163	124		39		0.427
II	12	8	(66.7)	4	(33.3)	
III	151	116	(76.8)	35	(23.2)	
Grade pre-CRT	154	116		38		0.515
Low	58	42	(72.4)	16	(27.6)	
Moderate-High	96	74	(77.1)	22	(22.9)	
ypT ^3^	163	124		39		0.026
0	25	24	(96)	1	(4)	
1	20	17	(85)	3	(15)	
2	57	38	(66.7)	19	(33.3)	
3–4	61	45	(73.8)	16	(26.2)	
ypN ^4^	163	124		39		0.005
N0	122	100	(82)	22	(18)	
N1	36	20	(55.6)	16	(44.4)	
N2	5	4	(80)	1	(20)	
Pathological stage	163	124		39		0.004
yp0	24	23	(95.8)	1	(4.2)	
ypI	62	47	(75.8)	15	(24.2)	
ypII	36	30	(83.3)	6	(16.7)	
ypIII	41	24	(58.5)	17	(41.5)	
Adjuvant treatment	163	124		39		0.085
No	37	24	(64.9)	13	(35.1)	
5-FU ^5^	94	77	(81.9)	17	(18.1)	
FOLFOX ^6^	28	19	(67.9)	9	(32.1)	
Other	4	4	(100)	0	(0)	

^1^ ECOG= Eastern Cooperative Oncology Group; ^2^ CRT= Chemoradiotherapy; ^3^ ypT= tumor size after CRT; ^4^ ypN= pathological lymph node after CRT; ^5^ 5-FU= 5-fluorouracil; ^6^ FOLFOX= Fluorouracil, Oxaliplatin, Leucovorin.

**Table 2:** Analysis of the potential value of miR-199b expression levels to predict pathological response to neoadjuvant CRT in LARC.

Responders vs. Non-Responders						
MiR-199b Expression	No. Cases	MiR-199b High (%)		MiR-199b Low (%)		*p*
Response	163	124		39		0.004
Non-Response ^1^	84	56	(45.2)	28	(71.8)	
Response ^2^	79	68	(54.8)	11	(28.2)	

^1^ Non-Response: poor or minimal pathological response; ^2^ Response: moderate or complete pathological response.

**Table 3:** Multivariate logistic analysis for pathological response in 163 LARC patients.

		Response ^1^ vs. Non-Response ^2^			
Parameter		OR ^4^	95% CI ^3^		*p*
			Lower	Upper	
Gender					0.976
	Male	1.000			
	Female	1.010	0.524 to 1.948		
Age					0.221
	<70	1.000			
	≥70	1.536	0.772 to 3.057		
Grade pre-CRT ^5^					0.149
	Low	1.000			
	Moderate-High	1.486	0.868 to 2.545		
Clinical stage					0.665
	II	1.000			
	III	0.870	0.465 to 1.631		
ECOG ^6^					0.646
	0	1.000			
	1–2	0.841	0.401 to 1.762		
MiR-199b					0.007
	High	1.000			
	Low	3.020	1.361 to 6.700		

^1^ Response: moderate or complete pathological response; ^2^ Non-Response: poor or minimal pathological response; ^3^ CI: confidence interval; ^4^ OR: Odds ratio; ^5^ CRT: chemoradiotherapy; ^6^ ECOG: Eastern Cooperative Oncology Group.

**Table 4:** Univariate and multivariate Cox analyses in the cohort of 163 LARC patients.

		Univariate OS ^1^ Analysis				Multivariate OS Cox Analysis			
Parameter		HR ^3^	95% CI ^2^		*p*	HR	95% CI		*p*
			Lower	Upper			Lower	Upper	
Gender					0.439				-
	Male	1.000							
	Female	0.785	0.426 to 1.448			-	-		
Age					0.018				0.013
	<70	1.000				1.000			
	≥70	2.062	1.123 to 3.758			2.138	1.171 to 3.904		
ypT ^4^					0.051				-
	0–2	1.000							
	3–4	1.306	0.998 to 1.710			-	-		
ypN ^5^					0.003				0.392
	N-	1.000				1.000			
	N+	2.498	1.355 to 4.605			1.409	0.642 to 3.095		
Pathological stage					0.013				0.257
	0–I	1.000				1.000			
	II–III	2.161	1.179 to 3.961			1.553	0.725 to 3.325		
ECOG ^6^					0.094				-
	0	1.000							
	1–2	1.660	0.918 to 3.002			-	-		
MiR-199b					<0.001				0.001
	High	1.000				1.000			
	Low	3.626	1.968 to 6.680			3.123	1.643 to 5.934		

^1^ OS: overall survival; ^2^ CI: confidence interval; ^3^ HR: Hazard ratio; ^4^ ypT: tumor size after chemoradiotherapy (CRT); ^5^ ypN: pathological lymph node after CRT; ^6^ ECOG: Eastern Cooperative Oncology Group.

**Table 5:** Association between miR-199b expression and recurrence in LARC patients.

MiR-199b Expression	No. Cases	MiR-199b High (%)		MiR-199 Low (%)		*p*
Recurrence	163	124		39		0.004
No	124	101	(81.5)	23	(18.5)	
Yes	39	23	(59)	16	(41)	
Place of elapse	31	18		13		0.543
Liver	3	2	(66.7)	1	(33.3)	
Lung	15	8	(53.3)	7	(46.7)	
Retroperitoneum	2	1	(50)	1	(50)	
Bone	2	2	(100)	0	(0)	
Peritoneum	1	0	(0)	1	(100)	
Abdominal wall	1	0	(0)	1	(100)	
Liver and lung	7	5	(71.4)	2	(28.6)	

## Data Availability

Data sharing is not applicable for this article.

## Supplementary Materials

The following are available online at https://www.mdpi.com/article/10.3390/cancers13195003/s1, Figure S1: Kaplan–Meier analysis showing the impact of miR-199b expression in time to metastatic development, Figure S2: Comparison of miR-199b expression levels in 24 paired pre and post-treatment samples from 24 LARC patients: (a) Box-plot showing global miR-199b expression changes, (b) Scatter plot showing miR-199 expression changes in each patient, Table S1: Evaluation of statistical associations between clinical and molecular parameters and miR-199b expression in 185 LARC patients, Table S2: Association between mir-199b expression and tumor size or lymph node positivity in 163 LARC patients, Table S3: Univariate and multivariate Cox analyses for EFS in the cohort of 163 LARC patients.
