# Supplementary material for: Validation of microRNA-199b as A Promising Predictor of Outcome and Response to Neoadjuvant Treatment in Locally Advanced Rectal Cancer Patients
Source: Cancers (Basel). 2021 Oct 5;13(19):5003. doi: 10.3390/cancers13195003 (PMC8507802; doi:10.3390/cancers13195003)
Supplement: Supplementary file 1 [file cancers-13-05003-s001.zip › Table S3.pdf]

Table S3. Univariate and multivariate Cox analyses in the cohort of 163 LARC patients.

	Univariate EFS[1] analysis				Multivariate EFS Cox analysis			
		95% CI[2]				95% CI		
	HR[3]	Lower	Upper	*p*	HR	Lower	Upper	*p*
Gender				0.492				-
Male	1.000							
Female	0.819	0.464 to 1.447			-	-		
Age				0.225				-
<70	1.000							
≥70	1.407	0.810 to 2.444			-	-		
ypT[4]				**0.020**				0.370
0-2	1.000				1.000			
3-4	1.371	1.051 to 1.789			1.153	0.845 to 1.574		
ypN[5]				**0.001**				0.311
N-	1.000				1.000			
N+	2.601	1.472 to 4.599			1.459	0.703 to 3.028		
Pathological stage				**0.003**				0.355
0-I	1.000				1.000			
II-III	2.392	1.343 to 4.259			1.457	0.657 to 3.231		
ECOG[6]				0.220				-
0	1.000							
1-2	1.427	0.808 to 2.521			-	-		
MiR-199b				**<0.001**				**0.010**
High	1.000				1.000			
Low	2.829	1.597 to 5.011			2.213	1.207 to 4.059		

[1]EFS: event-free survival; [2]CI: confidence interval; [3]HR: Hazard ratio; [4]ypT: tumor size after chemoradiotherapy (CRT); [5]ypN: pathological lymph node after CRT; [6]ECOG: Eastern Cooperative Oncology Group.
