# Supplementary material for: Validation of microRNA-199b as A Promising Predictor of Outcome and Response to Neoadjuvant Treatment in Locally Advanced Rectal Cancer Patients
Source: Cancers (Basel). 2021 Oct 5;13(19):5003. doi: 10.3390/cancers13195003 (PMC8507802; doi:10.3390/cancers13195003)
Supplement: Supplementary file 1 [file cancers-13-05003-s001.zip › Table S1.pdf]

Table S1. Evaluation of statistical associations between clinical and molecular parameters and miR-199b in 185 LARC patients.

	No. Cases	No. miR-199b high (%)	No. miR-199 low (%)	*p*
MiR-199b	185	144 (77.8)	41 (22.2)	
Age	185	144	41	0.491
<70	99	79 (79.8)	20 (20.2)	
≥70	86	65 (75.6)	21 (24.4)	
ECOG	185	144	41	0.343
0	124	94 (75.8)	30 (24.2)	
1-2	61	50 (82)	11 (18)	
Stage	185	144	41	0.902
I-II	19	15 (78.9)	4 (21.1)	
III	166	129 (77.7)	37 (22.3)	
