# Supplementary material for: Validation of microRNA-199b as A Promising Predictor of Outcome and Response to Neoadjuvant Treatment in Locally Advanced Rectal Cancer Patients
Source: Cancers (Basel). 2021 Oct 5;13(19):5003. doi: 10.3390/cancers13195003 (PMC8507802; doi:10.3390/cancers13195003)
Supplement: Supplementary file 1 [file cancers-13-05003-s001.zip › Table S2.pdf]

Table S2. Association between mir-199b expression and tumor size or lymph node positivity in 163 LARC patients.

	No. Cases	No. miR-199b high (%)		No. miR-199 low (%)		*p*
ypT	163	124		39		**0.007**
0-1	44	40	(32.3)	4	(10.3)	
>1	119	84	(67.7)	35	(89.7)	
ypN	163	124		39		**0.002**
N0	122	100	(80.6)	22	(56.4)	
N+	41	24	(19.4)	17	(43.6)	
